# The Role of Online Arts and Humanities in Medical Student Education: Mixed Methods Study of Feasibility and Perceived Impact of a 1-Week Online Course

**DOI:** 10.2196/27923

**Published:** 2021-09-22

**Authors:** Kaitlin Stouffer, Heather J Kagan, Margot Kelly-Hedrick, Julia See, Elizabeth Benskin, Suzy Wolffe, Philip Yenawine, Margaret S Chisolm

**Affiliations:** 1 Johns Hopkins University School of Medicine Baltimore, MD United States; 2 Department of Internal Medicine Johns Hopkins Hospital Baltimore, MD United States; 3 Duke University School of Medicine Durham, NC United States; 4 University of Miami School of Medicine Miami, FL United States; 5 The Baltimore Museum of Art Baltimore, MD United States; 6 Watershed Collective Baltimore, MD United States; 7 Department of Psychiatry and Behavioral Sciences Johns Hopkins University School of Medicine Baltimore, MD United States

**Keywords:** visual arts, professional identity formation, online, visual thinking strategies, arts and humanities, arts, humanities, education, medical students, medical education, teaching

## Abstract

**Background:**

The arts and humanities have been integrated into medical student education worldwide. Integrated arts and humanities courses have been found to serve four primary functions: mastering skills, perspective taking, personal insight, and social advocacy. To what extent and how arts and humanities programs achieve these educational outcomes remain unclear.

**Objective:**

In this study, we aimed to explore how the arts and humanities may lead to perceived benefits in clinical skills development, professional identity formation, and self-care, and to evaluate the feasibility of delivering an arts and humanities–based course online.

**Methods:**

We developed and delivered a 1-week online arts and humanities course to second- through fourth-year medical students. A total of 18 students enrolled in the course across its 2 offerings in Spring 2020. The course was primarily visual arts based but also included activities based in other arts and humanities, such as literature, reflective writing, dance, film, music, philosophy, and religion. Using a mixed methods approach, daily polls assessed student engagement in and perceptions of the various activities, and a postcourse survey assessed student perceptions of the course as a whole.

**Results:**

At least 93% of poll respondents (14/15 to 17/18) across the 2 cohorts rated each type of activity as good or excellent. Qualitative analysis of student responses to the postcourse survey revealed themes concerning both the form (overall course design and online format) and the function of the course (skills development, appreciation of new perspectives, and personal inquiry).

**Conclusions:**

Results suggested that the arts and humanities may support the development of clinically relevant skills and attitudes. A more unique finding was that integrative arts and humanities courses delivered online—including those that are primarily visual arts based—engage students and may yield personal and professional benefits.

## Introduction

The practice of medicine requires both bioscientific knowledge and humanistic caregiving. As the presence of science and technology in medicine expands, the scales increasingly tilt away from humanism to the detriment of patient care. The arts and humanities hold promise for righting that imbalance, and educators worldwide are calling to integrate arts and humanities programs across the continuum of medical education [1].

With regard to *what* the integration of the arts and humanities into medical education might achieve, the current literature highlights a number of diverse but related benefits. A previous review of the literature suggests that integrative arts and humanities programs serve three roles in medical education: additive, curative, and intrinsic. Art serves as a catalyst for reflection and discussion that can be enjoyable, enhance well-being, and support the development of clinically relevant skills, such as observation, communication, and clinical reasoning [2,3]. A more recent review proposes a slightly different conceptual model that suggests that, in addition to traditional skill mastery, the arts and humanities can promote perspective taking, personal insight, and social advocacy among medical learners [1,4].

A related concept, also thought to benefit from exposure to the arts and humanities [1], is professional identity formation (PIF), defined as a process of psychological and social development that occurs within the larger context of overall identity formation [5]. As part of PIF, medical learners must integrate reflection at an individual level and a collective level, iteratively rethinking and remolding their view of themselves as they reconcile their personal and professional identities [5].

With regard to *how* the arts and humanities might be effectively integrated, most programs have primarily used literature, reflective writing, or narrative medicine to teach medical students [1]. The few programs that focus on visual arts to teach clinical skills development are administered to preclinical medical students [6]. No visual arts–based program—either online or in-person—specifically designed for clinical-year medical students has been described in the literature.

To address this gap, we developed an in-person visual arts–based elective designed specifically for fourth-year medical students prior to entry into residency. We aimed the course primarily at PIF, with secondary considerations of clinical skills development and self-care, as clinical skills development is directly relevant to one’s development as a professional. Furthermore, support of self-care can help address the anxiety associated with reflection and compromise in PIF [5]. To meet the needs of our university’s students due to the COVID-19 pandemic, in March 2020 our team restructured this planned 4-week in-person course to a 1-week online course offered to second- through fourth-year medical students. We delivered this 1-week online course twice in Spring 2020.

Through this course, we sought to evaluate the feasibility of online engagement and to explore to what extent the arts and humanities, and particularly the visual arts, may facilitate clinical skills development, PIF, and self-care. In addition, we sought to understand which pedagogical strategies might best support engagement with the arts and humanities, using the unique circumstances of the COVID-19 pandemic to assess the feasibility or benefit of an online format. We hypothesized that students would engage in an online course and perceive growth in clinical skills, PIF, and self-care. We further hypothesized that participation in the course would lead students toward a clearer understanding of and a deeper appreciation for the role of arts and humanities in medical education.

## Methods

### Course Overview

In consultation with three museum educators (authors EB, SW, PY), the course was developed by a multidisciplinary team, comprised of a Johns Hopkins University (JHU) medical school faculty member and physician (author MSC), an internal medicine resident (author HJK), a medical student (author KS), and a research coordinator (author MKH). Two of the museum educators (EB and SW) were instrumental in developing the in-person activities, which the teaching team (MSC, HJK, KS, MKH, PY) adapted to an online format with the assistance of the third museum educator (PY) [7].

This 5-day full-time (40 hours/week of total expected work) online course was offered as an elective to second- through fourth-year JHU medical students in April and May 2020. The course consisted of five 2-hour synchronous online Zoom sessions each morning (Monday to Friday), followed by 6 hours of out-of-class assignments, which the students completed independently each afternoon. Each synchronous session followed the same structure: daily check-in, a visual thinking strategy (VTS) session, sharing of the previous day’s independent assignments, a unique daily group activity, an individual written reflection, and a closing meditation. VTS sessions were led by PY, co-developer of the VTS method and curriculum. In this method, the facilitator leads students in discussion of a particular object of observation—most frequently a piece of art—using a series of three questions: (1) What’s going on in this picture? (2) What do you see that makes you say that? and (3) What more can we find? [7]*.* In a randomized controlled trial with nonmedical learners, the VTS method has been shown to improve critical thinking skills and has been incorporated in a wide variety of educational settings among a spectrum of child to adult learners [8]. Other activities were facilitated by one or more members of the teaching team, with breakout rooms accommodating the variety of formats for these activities (Table 1).

**Table 1:** Synchronous session course structure.

Activities	Day 1	Day 2	Day 3	Day 4	Day 5
Daily check-in (5 min)	Same each day	Same each day	Same each day	Same each day	Same each day
First group activity (35 min)	Pair and Share Introduction	VTS^a^ session (*Interior, Mother and Sister of the Artist*, Édouard Vuillard)	VTS session (*St Hugh in the Carthusian Refectory*, Francisco de Zurbarán)	VTS session (*Wedding Portrait*, Njideka Akunyili Crosby)	VTS session (*Love After Love*, Derek Walcott)
Sharing from the previous day’s independent assignments (35 min)	N/A^b^	Object of Connection: Students find an object in their space that connects them to their family or community.	Relationship Gift: Students create a gift out of objects at home or in nature for the relationship described in the previous day’s group activity.	Motivations and Aspirations: Students select and reflect on an image from a collected set that resonates with their motivations and aspirations in the field of medicine.	Forest Bath: Students take a mindful walk through nature.
Unique large group activity (30 min)	VTS session (*Christina’s World*, Andrew Wyeth)	Relationship Activity: Students choose an image from a collected set and describe the relationship between two figures in the image.	Group Poem: Students are divided into two groups. Each member writes what the figure in the image is thinking or saying. The group constructs a poem out of each member’s phrase and performs the poem for the other group.	Video (*Martyrs*, Bill Viola)	Self-Care Personal Responses Tour: Students choose an image from a collected set in response to individual, unique prompts oriented toward self-care.
Individual written reflection (15 min)	Prompt: “Describe yourself through glasses of a different prescription than your own.”	Prompt: “Write about a family member you’ve never met.”	Prompt: “Write about the world within you.”	Prompt: “Describe the weight of the day.”	Prompt: “Write about what you hope is in that package, lies beneath that shell, or is written on the next page.”
Closing meditation (5 min)	*The Guesthouse*, Jellaludin Rumi	*Mercy Now*, Mary Gauthier	*One Art*, Elizabeth Bishop	*December 25*, Ted Kooser	*Lead*, Mary Oliver

^a^VTS: visual thinking strategy.

^b^N/A: not applicable.

Daily postsynchronous session independent assignments included online videos (eg, “The Danger of a Single Story,” TED talk by Chimamanda Ngozi Adichie, “Pina” by Wim Wenders), podcasts (eg, “A Poem About What Grounds You” from Poetry Unbound), readings (eg, “The Moral Urgency of Anna Karenina: Tolstoy’s Lessons for All Time and for Today” by Gary Saul Morson, “A Small Good Thing” by Raymond Carver, “The Hollow Men” by T.S. Eliot), a mindful walk through nature, and creating activities (eg, finding an object in their current space that reminds them of home, creating a gift for a relationship they observed in a chosen image) about which the group debriefed together the next day.

### Evaluation Methods

All 18 students who enrolled in the 2 offerings of the course (10 and 8, respectively) were introduced to the optional research study at the beginning of the course and invited to participate via daily polls and a postcourse survey. This research study protocol was reviewed and deemed exempt by the JHU School of Medicine Institutional Review Board (IRB00244745).

### Quantitative Measures

To assess real-time engagement in the online course, at the end of each synchronous session, we asked the students to rate the day’s synchronous and the previous day’s independent assignments and closing meditation on a 5-point Likert scale (1=poor to 5=excellent). Polls were anonymous, and we informed the students that participation was optional. The previous day’s independent assignments and closing meditation were evaluated 4 times due to the retrospective timing of these events, whereas all other activities were evaluated 5 times.

### Qualitative Measures

We conducted simple thematic analysis of the open-ended items of our survey. At the end of the last day of the course, we invited students to complete an anonymous 9-item Qualtrics survey soliciting their opinions on overall course execution (ie, choice and format of activities) and perceptions of any benefits of the course. To assess the latter, we asked students to complete the prompts “I used to think . . .” and “Now I think . . .” with regard to their view on the arts’ role in PIF, self-care, and clinical skills development (see Multimedia Appendix 1, which contains the complete survey). A total of 7 students in April (response rate=70%) and 4 students in May (response rate=50%) participated in the survey (11/18, 61% overall response rate).

To evaluate responses to the open-ended survey items, we followed a phasic method of coding and analysis [9]. First, two reviewers (HJK and KS) independently generated themes of surveys from cohorts 1 and 2 using open coding. Initial codes were then consolidated for each survey with the help of a third reviewer (MSC), and the two codebooks were merged into one final codebook. HJK and KS then independently recoded the surveys with NVivo software (QSR International, Doncaster, Australia) using the final codebook [10]. Final coding discrepancies were resolved with the third coder.

## Results

### Quantitative Measures

**Figure 1:**
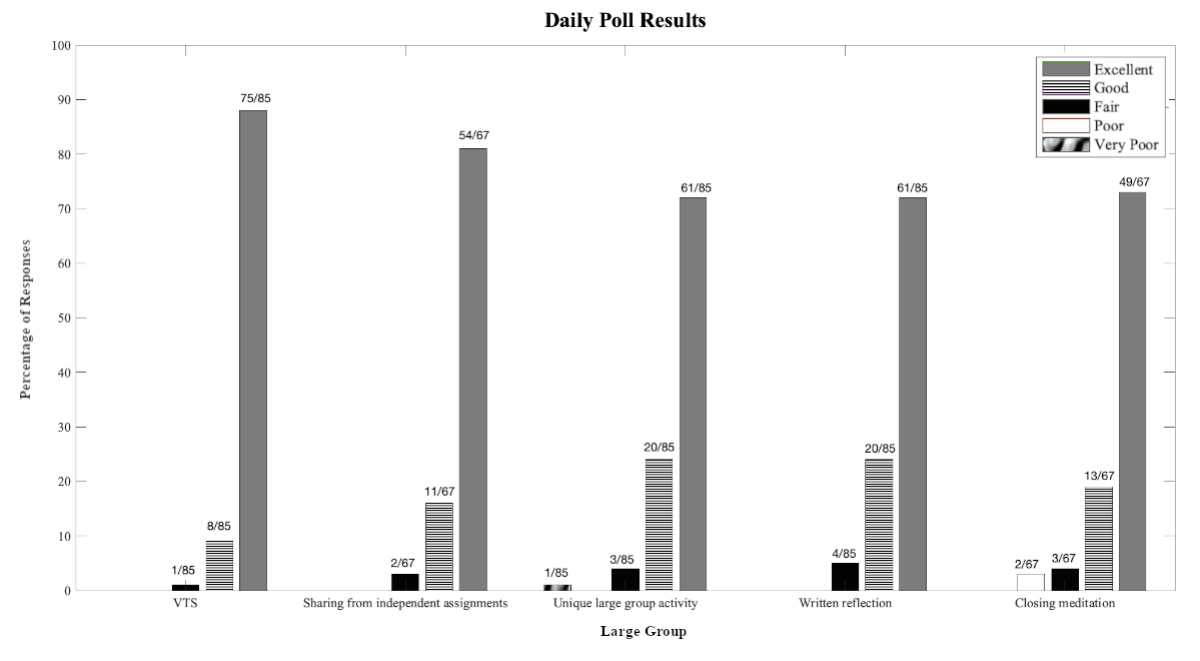
Daily poll results from cohorts 1 and 2 (N=8-10 and N=6-8 per day, respectively) tallied across 5 days. Percentages taken over the total number of responses per activity across 5 days across 2 cohorts. VTS: visual thinking strategy.

We report results of the daily polls in Figure 1. Results reflect average ratings across all 5 days of the course, with items evaluated 4 or 5 times over the course. At least 75% of students in each session (9/10 and 6/8 students in cohorts 1 and 2, respectively) participated in the polls each day, and the total participation across the 2 sessions summed to between 83% (15/18) and 100% (18/18) each day with an average of 94% (17/18) participation across the week. At least 93% (between 14/15 and 17/18) of respondents rated all activities as good or excellent. The most highly rated activities were VTS sessions and debriefs/discussions of the previous day’s assignments.

### Qualitative Measures

We report results of the postcourse survey in Table 2. From the survey, 5 themes emerged, which we grouped into 2 broad categories: form and function.

**Table 2:** Emergent themes from survey responses across cohorts 1 and 2 (n=11).

Theme		Description/content	Exemplary quote(s)
**Form**			
	Course design	Course activities Format of activities (synchronous vs asynchronous, large vs small group) Sequence of activities Facilitation methods	“In the spirit of recognizing how various activities and actions build on each other in subtle ways to make a big difference, I felt that each day was complementary to and enhanced the previous day’s work.”
	Online format	Aspects uniquely specific to the virtual platform	“The comfort of home can also facilitate reflection. Although there are benefits to being in a museum, being at home comes with unique advantages for self-reflection.”
**Function**			
	Skills development	Building of clinical skills: Observation Interpretation Clinical reasoning Empathy Communication	“It can help me to have an open mind, to observe, to listen.” “We can . . . come up with different interpretations [of art] like [a] differential diagnosis.”
	Appreciation of new perspectives	Exposure to diverse perspectives of others Understanding and appreciation of the importance of multiple perspectives in meaning making	“. . . each person’s perspective (including my own) is valid and enables us to see more; there are no right or wrong answers.”
	Personal inquiry	New and rediscovery of aspects of self Personal growth Inner peace Personal and professional identity integration Expansion of personal perspective Broadened view of the role of art in one’s personal life and profession	“The synthesis of all of this work left me feeling more mindful and human.” “It really helped me get in touch with myself and remember why I wanted to become a physician, which I feel is something I had lost recently.” “I feel that art can help me connect with the core feelings that led me to this profession and guide my career goals. Thus, art can help facilitate professional identity formation.”

#### Form

Course design and online format themes encompass insights into how structural elements of the course facilitate learning.

##### Course Design

This theme emerged from comments related to the sequence and facilitation methods of specific activities. Students appreciated beginning each synchronous session with a VTS session, describing it as a way to “get creative juices flowing.” Students enjoyed the interactive nature and varied format of the activities, facilitated via the use of breakout rooms.

##### Online Format

This theme emerged from comments regarding the advantages and disadvantages of an online platform. Some students cited physical distance as a “barrier to speak and share.” Others said “the comfort of home . . . facilitate[d] self-reflection” and allowed them to “share and overcome fears of speaking to a group.”

#### Function

Skills development, appreciation of new perspectives, and personal inquiry themes focused on the role the course played more broadly in the students’ professional and personal lives.

##### Skills Development

This theme emerged from comments highlighting the development of concrete skills applicable outside of the course.

Students felt that skills of empathy, observation, listening, and communication were strengthened through the course, transferrable “tools” they could “practice on their own time or with another group.” Students observed that discussions around art paralleled diagnostic reasoning and could help them “form a differential diagnosis.”

##### Appreciation of New Perspectives

This theme emerged from comments related to the importance of others’ perspectives in shaping one’s own understanding, recognizing bias, and building something larger than themselves.

Through group activities and discussions, students became “more aware” of the many interpretations that coexist around a single piece of art. They noticed the “power of observation as a group” and of multiple perspectives in prompting the “acceptance of uncertainty/ambiguity” and recognition of their own biases and “pre-existing thoughts.” Students welcomed the opportunity to “broaden their perspectives” and praised the summative perspective that resulted from teamwork as “something greater than each on [their] own.”

##### Personal Inquiry

This theme emerged from comments describing personal growth and a deepened connection with self.

Students “learned more about [themselves],” reflected on their growth in medical school, and “rediscovered parts of [themselves],” such as their motivations for pursuing medicine. They achieved an “inner peace” and a sense of “being in touch with oneself.” The course “built character” and “made [them] a better person.” Students realized a sense of ownership over their development, recognizing how they, not others, “need to be the one[s] to form [their] professional identity.”

Students noted shifts in their views of the meaning of art and its role in their lives. They deemed art a means of self-expression, a teaching tool, an active rather than a passive activity, and a discussion-based group experience rather than an individual one. They cited art as a platform for “approaching difficult topics like death.” Students considered art “essential” to their PIF, allowing them to “connect with [themselves]” and others and cultivate self-care practices.

##### Complex Comments

We also identified a number of complex comments embodying multiple themes. Intersecting both personal inquiry and an appreciation of perspectives, one student wrote, “Arts have an important role in clinical skills development—VTS [sessions] helped [me] recognize the limitations of [my] own views and cultivate an appreciation for others’ views.” Another student encompassed all three functional categories in writing that “a sense of community and connection to others . . . can enhance one’s professional identity and connect with others in [the] field.”

## Discussion

### Principal Findings

We explored the role of an online, primarily visual arts–based arts and humanities course designed to facilitate clinical skills development, PIF, and self-care in 2 cohorts of medical students. From student responses to open-ended survey items, we identified 2 formal themes (course design and online format) and 3 functional themes (skills development, appreciation of new perspectives, and personal inquiry). Consistent with a recent review of online medical education endeavors [11], our results suggest an online platform can be an effective format for integrative arts and humanities programs, particularly visual arts–based courses. Additionally, in supporting self-care, our course addresses the need for more holistic approaches in medical education in order to combat the impact on students of not only the usual stresses medical education and training in general hold but also those additional stresses experienced by students during the COVID-19 pandemic [11].

The functional themes that emerged from our study reflect elements of the major conceptual models regarding integrative arts and humanities programs [1,2,4]. Overall, the similarities in themes that emerged from our study to those of models described previously in the literature suggest the strength and validity of these models’ conceptualization of the role of integrative arts and humanities programs in skills, relational, and personal development. This congruence—coupled with complex statements that reflect multiple themes—highlights that benefits of integrative arts and humanities programs are multifaceted and interwoven, and, as a result, can be difficult to measure.

Our results offer several additional insights into how arts and humanities programs can be effective. First, the results suggest that it is feasible to deliver arts and humanities programs online—even if primarily visual arts based—in a way that engages students. This expands upon the previously identified role of visual arts–based courses solely in skills development and supports the use of such courses in other educational avenues as well [12]. Second, our results suggest that even brief arts and humanities programs, such as this 1-week course, can lead to benefits. Third, arts and humanities programs may offer benefits to both preclinical- and clinical-year students. Fourth, integrative arts and humanities programs have the potential to inspire future growth and development, both personally and professionally. Fifth, delivering arts and humanities programs in an interactive group format may be especially important in fostering skills mastery, perspective taking, and personal insight among students. In many ways, the structure of VTS sessions parallels the process of forming a clinical diagnosis. In VTS sessions, students are asked to (1) make observations over time, (2) base inferences on evidence, (3) listen to others’ ideas, (4) hold multiple interpretations as plausible, and (5) revise their interpretations, often tolerating ambiguity. As such, the VTS method supports the development of observational, communication, interpersonal, and diagnostic skills (1,2,3); of perspective taking (3,4); and of personal insight (5) in students as they reflect on their own views.

### Limitations

Although our study supports a role for online arts and humanities programs in medical student education that is consistent with previous studies, we acknowledge several limitations. First, like all studies, ours was vulnerable to bias. Yet, to attempt to mitigate bias, we conformed to a standard methodology for our qualitative analysis, with two independent reviewers and the use of a third reviewer for resolving discrepancies. Second, our course enrollees were a self-selected sample from a single institution, and thus, their experience of the course may not be generalizable to other students. Third, although our daily poll and postcourse survey response rates were greater than 50%, our total class enrollment (N=18) and subsequent sample size (n=11, 61%) were both small. Fourth, our methods of evaluation were limited to one survey administered one time (at the end of the course) and, therefore, could not assess (1) potential longitudinal benefits of our course, (2) discrete changes in skills or behavior, or (3) more extensive sentiments as might have been gleaned through measures such as interviews or focus groups. Fifth, although the course faculty was interprofessional, including learners, our museum educator facilitator (PY) who led the VTS sessions is a cofounder of the method and thus the impact of the VTS activity may not be generalizable to other teaching teams. Sixth, as the course was delivered during the COVID-19 pandemic, this unusual circumstance may have exaggerated the educational benefits of online arts and humanities programs compared to pre-pandemic times.

### Conclusions

Amid the COVID-19 pandemic, we transitioned an in-person art museum–based course aimed at fourth-year medical students to an online platform. We aimed to quantitatively and qualitatively explore whether and how the course engaged students and influenced clinical skills development, PIF, and self-care. In addition, we aimed to understand which activities were perceived by the students as most impactful in achieving these results. Our study highlighted the role of a diverse set of activities, including visual arts–based activities, such as VTS sessions, in engaging students and potentially fostering development in areas of skills development, appreciation of new perspectives, and personal inquiry. Additionally, we demonstrated that these activities could be engaging and perceived as beneficial even when delivered online. Overall, our findings support the Association of American Medical Colleges’ recommendations for investigating and implementing arts and humanities integration into medical education [1]. To accurately capture the full breadth and depth of the impact of the arts and humanities in medical student teaching, continued evaluation of such programs, including controlled trials, learner assessment at the level of behavioral change, longitudinal follow-up, and ongoing revision of current conceptual models [1], will be needed.

## Appendix

Multimedia Appendix 1 Postcourse survey administered to students.

## Abbreviations

JHU: Johns Hopkins University
PIF: professional identity formation
VTS: visual thinking strategy
