# Supplementary material for: The Role of Online Arts and Humanities in Medical Student Education: Mixed Methods Study of Feasibility and Perceived Impact of a 1-Week Online Course
Source: JMIR Med Educ. 2021 Sep 22;7(3):e27923. doi: 10.2196/27923 (PMC8495565; doi:10.2196/27923)
Supplement: Multimedia Appendix 1 [file mededu_v7i3e27923_app1.pdf]

Summative Survey – Feedback administered as Qualtrics survey on last day of course

We would like to get your overall feedback on the entire 1-week course.

Looking back on the course, which activity was the most meaningful to you and why? [text box]

Reflecting back on your thinking prior to taking the course and now at the end of the course, please complete the following statements:

Regarding the role of the arts in **professional identity formation,** I used to think [textbox], now I think [textbox]

Regarding the role of the arts in **clinical skills development**, I used to think [textbox], now I think [textbox]

Regarding the role of the arts in **self-care**, I used to think [textbox], now I think [textbox]

If you have any comments about the **online format** of the course (strengths and/or weaknesses), please share them here:

Do you have any suggestions for improving the course in any way?

Have you engaged in any other arts-based experience as part of your medical school curriculum (formal or informal) at Johns Hopkins (prior to this course)?

- o Yes
- o No

Please explain [textbox]

Would you support the creation of a formal program to integrate arts-based experiences into the medical school curriculum at Johns Hopkins?

- o Yes
- o No

If yes the following question appear:

Format of course (select one):

- o I would prefer an **entirely online** format.
- o I would prefer an **entirely in-person** format
- o I would prefer a **mix of in-person and online** formats

If they selected the 2nd or 3rd response to the previous question:

Which type of in-person format would you be interested in? (Select all that apply)

On the medical campus

Off the medical campus (e.g., at an art museum)

Length of course:

I would prefer a [drop down with options: 1-week, 2-week, 3-week, 4-week] course.

Would you choose to take a TIME course with art museum-based experiences?

- Yes
- No
- Maybe

If yes the following question appear:

Format of course (select one):

- I would prefer an **entirely online** format.
- I would prefer an **entirely in-person** format
- I would prefer a **mix of in-person and online** formats

If they selected the 2nd or 3rd response to the previous question:

Which type of in-person format would you be interested in? (Select all that apply)

On the medical campus

Off the medical campus (e.g., at an art museum)

Please select one whether you would like your responses to be included in the research study. As a reminder, your decision will not affect grades or standing at Johns Hopkins School of Medicine.

- I **<u>AGREE</u>** to having my responses to this survey included in a research study evaluating this course. I understand my responses will be anonymous and not linked to my name or any other identifying information. Aggregate data will be shared with other researchers and the public via presentations and/or publications.

- I **<u>DO NOT AGREE</u>** to having my responses included in a research study evaluating this course. I understand this means my responses will only be reviewed by the course instructors to improve the course in the future.

Thank you for completing the evaluation survey!
